# Elevated Adsorption of Lead and Arsenic over Silver Nanoparticles Deposited on Poly(amidoamine) Grafted Carbon Nanotubes

**DOI:** 10.3390/nano12213852

**Published:** 2022-11-01

**Authors:** Gururaj M. Neelgund, Sanjuana F. Aguilar, Mahaveer D. Kurkuri, Debora F. Rodrigues, Ram L. Ray

**Affiliations:** 1 Department of Chemistry, Prairie View A&M University, Prairie View, TX 77446, USA; 2 Centre for Research in Functional Materials (CRFM), JAIN University, Jain Global Campus, Bengaluru 562112, Karnataka, India; 3 Department of Civil and Environmental Engineering, University of Houston, Houston, TX 77004, USA; 4 College of Agriculture and Human Sciences, Prairie View A&M University, Prairie View, TX 77446, USA

**Keywords:** carbon nanotubes, lead, arsenic, adsorption, poly(amidoamine), silver nanoparticles

## Abstract

An efficient adsorbent, CNTs–PAMAM–Ag, was prepared by grafting fourth-generation aromatic poly(amidoamine) (PAMAM) to carbon nanotubes (CNTs) and successive deposition of Ag nanoparticles. The FT–IR, XRD, TEM and XPS results confirmed the successful grafting of PAMAM onto CNTs and deposition of Ag nanoparticles. The absorption efficiency of CNTs–PAMAM–Ag was evaluated by estimating the adsorption of two toxic contaminants in water, viz., Pb(II) and As(III). Using CNTs–PAMAM–Ag, about 99 and 76% of Pb(II) and As(III) adsorption, respectively, were attained within 15 min. The controlling mechanisms for Pb(II) and As(III) adsorption dynamics were revealed by applying pseudo-first and second-order kinetic models. The pseudo-second-order kinetic model followed the adsorption of Pb(II) and As(III). Therefore, the incidence of chemisorption through sharing or exchanging electrons between Pb(II) or As(III) ions and CNTs–PAMAM–Ag could be the rate-controlling step in the adsorption process. Further, the Weber–Morris intraparticle pore diffusion model was employed to find the reaction pathways and the rate-controlling step in the adsorption. It revealed that intraparticle diffusion was not a rate-controlling step in the adsorption of Pb(II) and As(III); instead, it was controlled by both intraparticle diffusion and the boundary layer effect. The adsorption equilibrium was evaluated using the Langmuir, Freundlich, and Temkin isotherm models. The kinetic data of Pb(II) and As(III) adsorption was adequately fitted to the Langmuir isotherm model compared to the Freundlich and Temkin models.

## 1. Introduction

Lead and arsenic are known for their toxicity and are widely distributed in the environment, particularly in water sources [1]. These elements, under low concentration, can also be hazardous to aquatic and non-aquatic creatures and plants. Lea and arsenic are carcinogenic, mutagenic, and teratogenic [2,3]. The ingestion of these elements can cause severe adverse effects that include hypertension, neurological complications, cardiovascular disease, intestinal disorders, hematopoietic dysfunction, mental impairment, organ failure, and malfunctioning of the immune and reproductive systems [4,5,6,7,8,9]. Lead and arsenic are listed as global priority pollutants because of their high toxicity, stability, and non-biodegradability [10]. It has been reported that worldwide more than 200 million people have been affected by the consequences of these elements [4]. The primary source of exposure to these lethal elements is water through regular activities like drinking, cooking, and irrigation. Water sources are getting contaminated with arsenic by both natural processes and anthropogenic activities like weathering processes, geochemical reactions, biological activities, combustion of fossil fuels, volcanic eruptions, mining activities, leaching of artificial arsenic compounds, smelting of metals ores, desiccants, wood preservatives, agricultural pesticides, and related anthropogenic activities [11,12,13,14]. In nature, arsenic exists in organic and inorganic forms. However, the organic form of arsenic is not of importance as it undergoes biotransformation and detoxifies through methylation [15]. At the same time, the inorganic form of arsenic exists in four oxidation states viz., −3, 0, +3, and +5. Out of these, −3 and 0 oxidation states are scarce, so +3 and +5 usually exist in water. Nevertheless, the presence of either a +3 or +5 state of arsenic depends on the redox and pH conditions of water [15,16]. Accordingly, trivalent arsenite, As(III), and pentavalent arsenate, As(V), commonly occur in water. In particular, As(III) transpires in reducing conditions, and As(V) exists in oxidizing conditions of water [4]. However, both As(III) and As(V) species are highly toxic and non-biodegradable [6]. Comparatively, As(III) is more highly mobile and stable than As(V) due to its stable electronic configuration [11]. In addition, As(III) has higher cellular uptake ability and binding affinity to vicinal sulfhydryl groups that react with various proteins and inhibit their activity [6,11,17]. In specific, As(III) is about sixty times more toxic than As(V) [6].

Another toxic element, lead, contaminates the water through industrial and anthropogenic sources. The primary sources of information pollution are industries such as battery manufacturing, printing, fuels, photographic materials, ceramic, glass, and explosive manufacturing [18]. In the atmosphere, lead persists as bivalent, Pb(II). It is also likewise toxic as As(III) and widely distributed. Because of their toxicity and detrimental effects, Pb(II) and As(III) are unsafe, specifically their association with water. Therefore, eliminating these toxic components present in water is a mandatory need. For the removal of toxic Pb(II) and As(III), several methods like chemical precipitation [19], ion exchange [20], membrane technology and reverse osmosis [21], electro-dialysis [22], and adsorption [23] have been developed. Nevertheless, chemical precipitation makes it difficult to remove low-concentrated arsenic like 10 mg/L from water [24]. In addition, chemical precipitation is less effective in the removal of As(III), and it requires the pre-oxidative conversion of As(III) to As(V) during its abolition [25]. Similarly, ion exchange is less effective in removing As(III), and the development of ion exchange resins is expensive [24]. Additionally, the membrane technology-aided reverse osmosis is costly, requiring external pressure to pass the contaminated water through the membrane. Besides, the discharge of the concentrate, membrane fouling, and flux decline is inevitable in reverse osmosis [26]. In electro-dialysis, several insoluble coagulants generate and deposit over the cathode [27]. Overseeing these drawbacks, the better alternative and promising technique for removing Pb(II) and As(III) is adsorption. Adsorption is a facile, efficient, accessible, low-cost, low-energy technique [28]. Additionally, using the adsorption technique, it is possible to remove trace amounts of Pb(II) and As(III) from water. Overall, adsorption has played a significant role in eliminating various contaminants from water. Consequently, due to its exceptional benefits, adsorption is a gifted technique for efficiently removing Pb(II) and As(III).

In the process of adsorption, selecting an adsorbent is a vital step in the success of the process. For this purpose, insoluble solid materials with high specific surface area and active functional groups are elected adsorbents. In this context, several adsorbents have been developed and tested for their efficiency in removing different pollutants [6,12,29]. The dynamic adsorbents are based on activated carbon [30], activated alumina [31], inorganic minerals [32], biomass adsorbents [33,34,35,36,37,38], polymer [39,40,41], carbon nanotubes (CNTs) [42,43,44] graphene oxide [45,46], metal-organic frameworks [47], microplastics [48] and more. Among these, CNTs based adsorbents are particularly interesting because their unique physical and chemical properties are relevantly suitable for adsorption [42,43,44]. During the CNTs, aided adsorption process, strong interactions between CNTs and pollutants alter contaminants’ mobility, bioavailability, and environmental risk [44]. The high surface area, well-defined structures, and uniform surfaces of CNTs facilitate the adsorption mechanism and process. Abundant active sites and functional groups exist over CNTs supporting the adsorption process. Furthermore, adsorption over CNTs transpires with different mechanisms, viz., ion exchange, coordination interaction, electrostatic interaction, and physical adsorption, which augment the adsorption [44]. Beyond its exceptional properties, the employment of CNTs in adsorption technology is still fragmentary, so further exploration is required. The main hindrance that restrained the application of CNTs in adsorption technology is their hydrophobic nature, which reduces the adsorption rate. However, the functionalization of CNTs introduces reactive functional groups over their surface that can significantly increase their selectivity and sensitivity toward pollutants [43]. In this fashion, CNTs can be modified to be hydrophilic for the adsorption of pollutants and more reactive by functionalizing with secondary materials. The functionalization of CNTs generates chemically active sites around defective segments such as pentagons, oriented against a tube body which generally consists of only hexagons; and it may be this that gives CNTs their remarkable ability to interact with pollutants [43,49]. Accordingly, the functionalization of CNTs is the critical step to improving their absorption efficiency. For the functionalization of CNTs, dendritic polymers are particularly interesting because of their unusual structure and properties [50]. Among different dendrimers, poly(amidoamine) (PAMAM) is predominantly interesting because of its symmetrical structure, controllable molecule chains, vast internal cavities, and abundant functional groups [51]. Based on these special structural characteristics, PAMAM persists with unique properties, such as high hydrophilicity, high dispersing ability, high bio-affinity, and ease of modification [51]. Therefore, PAMAM is especially interesting for the adsorption of heavy metal ions and plays an important role in the functionalization of nanomaterials [51,52,53,54]. A large number of amino and amide functional groups of PAMAM dendrimers can strongly chelate heavy metal ions, thus improving the enrichment efficacy [51,52,53,54]. Considering its importance, herein, CNTs were functionalized by grafting with fourth-generation aromatic PAMAM and sequential deposition of Ag nanoparticles. Thus produced, CNTs–PAMAM–Ag was explored in evaluating the adsorption efficiency of two important toxic pollutants, Pb(II) and As(III). The controlling mechanisms and dynamics of the adsorption of Pb(II) and As(III) over CNTs–PAMAM–Ag were estimated by implementing the pseudo-first and second-order kinetic models. The reaction pathways and the rate-controlling step in adsorption were evaluated using Weber–Morris intraparticle pore diffusion model. Furthermore, the adsorption equilibrium was estimated by fitting the experimental results with Langmuir, Freundlich, and Temkin isotherm models.

## 2. Experimental

### 2.1. Materials

CNTs prepared by the CVD process were received from Carbon Nanotechnology Laboratory at Rice University, Houston, TX, USA. All the chemicals were purchased from Millipore-Sigma (St. Louis, MO, USA) and used as received. The aqueous solutions were prepared using ultrapure water obtained by the Milli-Q Plus system (Millipore, Burlington, MA, USA).

### 2.2. Preparation of CNTs–PAMAM–Ag

The CNTs–PAMAM–Ag was prepared using the reported method [55]. In brief, 100 mg of CNTs-PAMAM, obtained by Michael’s addition process, were dispersed in 15 mL of DI water by sonication, and a 10 mL aqueous solution of AgNO_3_ (0.01 mol/L) was slowly added. The resulting suspension was allowed to stir under ambient conditions for 8 h and centrifuged. Thus, formed CNTs–PAMAM–Ag was purified by successive washings with DI water and ethanol and dried under vacuum. For control experiments, hydrophobic nature pristine CNTs were modified to hydrophilic oxidized CNTs, and PAMAM was prepared using Michael’s addition procedure [55].

### 2.3. Adsorption Experiments

The stock solution of Pb(II) and As(III) with a 1 g/L was prepared in DI water using lead(II) nitrate and sodium arsenite, respectively. Further, the stock solution was diluted to desired concentrations using DI water. The kinetic adsorption experiments of Pb(II) and As(III) were performed to evaluate the contact time required to attain equilibrium. In the typical experiment, 100 mg of adsorbent was dispersed into 500 mL of Pb(II) and As(III) solution with a concentration of 40 μg/L with an initial pH of 7.67 and 8.11, respectively. Then, the mixtures were allowed to stir at room temperature, and an adequate quantity of samples was collected after the required contact time. Successively, the adsorbent was separated by centrifugation, and the concentration of residual Pb(II) or As(III) in the solution was estimated by the atomic absorption spectrometer. The efficiency in adsorption of Pb(II) and As(III) as a function of time was monitored for 120 min. Then, the adsorbed quantity of Pb(II) and As(III) was evaluated using the following equation.

(1) $$q_{t}=\frac{(C_{0}-C_{t})\;V}{M}$$

where *q_t_* is the amount of pollutant adsorbed (mg/g) at time t; *C*_0_ is the initial concentration of contaminant in solution (mg/L), and *C_t_* is the concentration of pollutant in solution (mg/L) at time *t*; *V* is the volume of the solution (L), and *M* is the amount of adsorbent (g).

The efficiency of adsorbent in the removal of Pb(II) and As(III) was calculated by:

(2) $$Removal\;efficiency\;\left(\%\right)=\frac{(C_{0}-C_{t})\;V}{C_{0}}\times 100$$

For adsorption isotherms experiments, 10 mg of CNTs–PAMAM–Ag was added to 50 mL of Pb(II) or As(III) solution and allowed to stir at room temperature for 24 h to reach the equilibrium. To obtain the adsorption isotherms, the concentration of Pb(II) and As(III) solution was varied from 1 to 10 mg/L. After reaching the equilibrium, CNTs–PAMAM–Ag was separated by centrifugation, and the concentration of Pb(II) or As(III) in the solution was measured using the atomic absorption spectrometer. Then, the adsorption of Pb(II) or As(III) at equilibrium, *q_e_* (mg/g), was determined by:

(3) $$q_{e}=\frac{(C_{0}-C_{e})\;V}{M}$$

where *q_e_* is the amount of Pb(II) and As(III) adsorbed (mg/g) at equilibrium.

### 2.4. Effect of pH on Adsorption of Pb(II)

To evaluate the effect of pH on the adsorption of Pb(II). The pH of the Pb(II) solution was varied from 4 to 12 using 0.1 M HCl and NaOH solutions. Other parameters were kept constant.

### 2.5. Desorption and Reuse of Adsorbent

After the adsorption experiment of Pb(II), CNTs–PAMAM–Ag was collected by centrifugation, dispersed in 0.1 M HCl solution, and stirred at room temperature for 2 h. Then, the CNTs–PAMAM–Ag was separated by centrifugation, washed with DI water, and dried under vacuum. Thus recovered CNTs–PAMAM–Ag was employed in the next cycle of adsorption of Pb(II). To estimate the reusability of CNTs–PAMAM–Ag, four adsorption–desorption cycles were performed.

### 2.6. Characterization

The FT-IR spectra were acquired using Thermo-Nicolet IR 2000 spectrometer (Madison, WI, USA) with KBr, and the XRD were recorded on a Scintag X-ray diffractometer (Cupertino, CA, USA), model PAD X, equipped with a Cu Kα photon source (45 kV, 40 mA) at the scanning rate of 3 °/min. Transmission electron microscopy (TEM) images were obtained with the Hitachi H-8100 microscope (Tokyo, Japan) at 200 kV. The concentration of Pb(II) and As(III) was estimated by Varian SpectrAA 220FS (Lake Forest, CA, USA) atomic absorption spectrometer.

## 3. Results and Discussion

**Figure 1:**
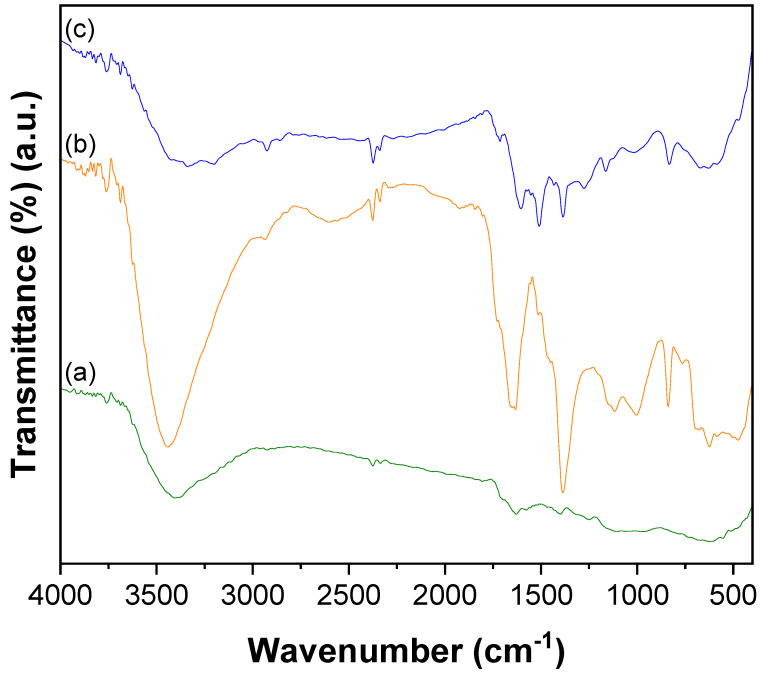
FT–IR spectra of (**a**) CNTs, (**b**) CNTs–PAMAM, and (**c**) CNTs–PAMAM–Ag.

The FT–IR spectrum of oxidized CNTs, presented in Figure 1a displayed the absorption bands of A2u and E1u phonon modes of CNTs at 611 and 1629 cm^−1^, respectively [56]. The broad feature appeared in the range of 3652 and 3000 cm^−1^ corresponding to the absorption of –OH stretching of carboxyl groups. The spectrum of CNTs–PAMAM (Figure 1b) demonstrated the characteristic absorption bands of CNTs and PAMAM. The peak displayed at 3441 cm^−1^ was related to N–H stretching frequency and the band found at 1631 cm^−1^ was due to carbonyl stretching of amide (–CO–NH). The bands that appeared at 2858 and 2934 cm^−1^ corresponded to symmetric and asymmetric stretching of –CH_2_, respectively. The bands revealed at 1515 and 837 cm^−1^ were by aromatic C–C and C–H para-aromatic out-of-plane vibrations, respectively. The bands found at 1003 and 1116 cm^−1^ was owing to aromatic –CH vibrations [55]. The bands of the aromatic ring displayed at 1515 (aromatic C–C), 1003 and 1116 (–CH), and 837 cm^−1^ (C–H para-aromatic out of plane vibration). The spectrum of CNTs–PAMAM–Ag (Figure 1c) exhibited the representative peaks of CNTs and PAMAM, however, the position of the peaks was slightly shifted, and the intensity of the bands was reduced.

**Figure 2:**
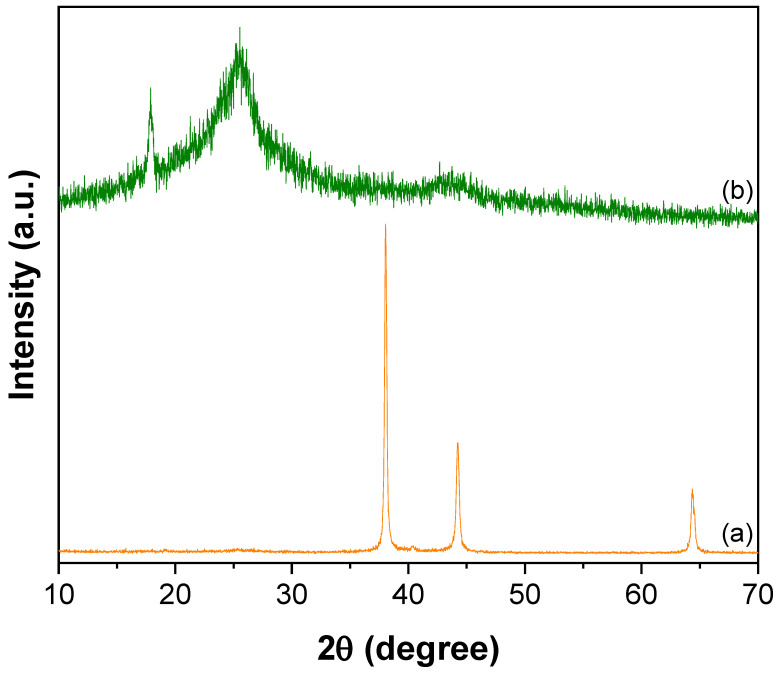
XRD pattern of (**a**) CNTs–PAMAM–Ag and (**b**) CNTs–PAMAM.

The powder XRD of CNTs–PAMAM–Ag, shown in Figure 2a, depicted the intense diffractions at 38.0, 44.2, and 64.3° corresponding to (1 1 1), (2 0 0), and (2 2 0) reflections of silver with face-centered cubic (fcc) symmetry, respectively [55,57,58]. The reflections of CNTs were not observed in CNTs–PAMAM–Ag (Figure 2a); however, these were distinctly found in CNTs–PAMAM (Figure 2b). The indistinct visibility of CNTs peaks in CNTs–PAMAM–Ag could be due to the effective exfoliation of CNTs, the significant intensity of Ag reflections, and adequate coverage of the surface of CNTs by densely populated deposition Ag nanoparticles.

**Figure 3:**
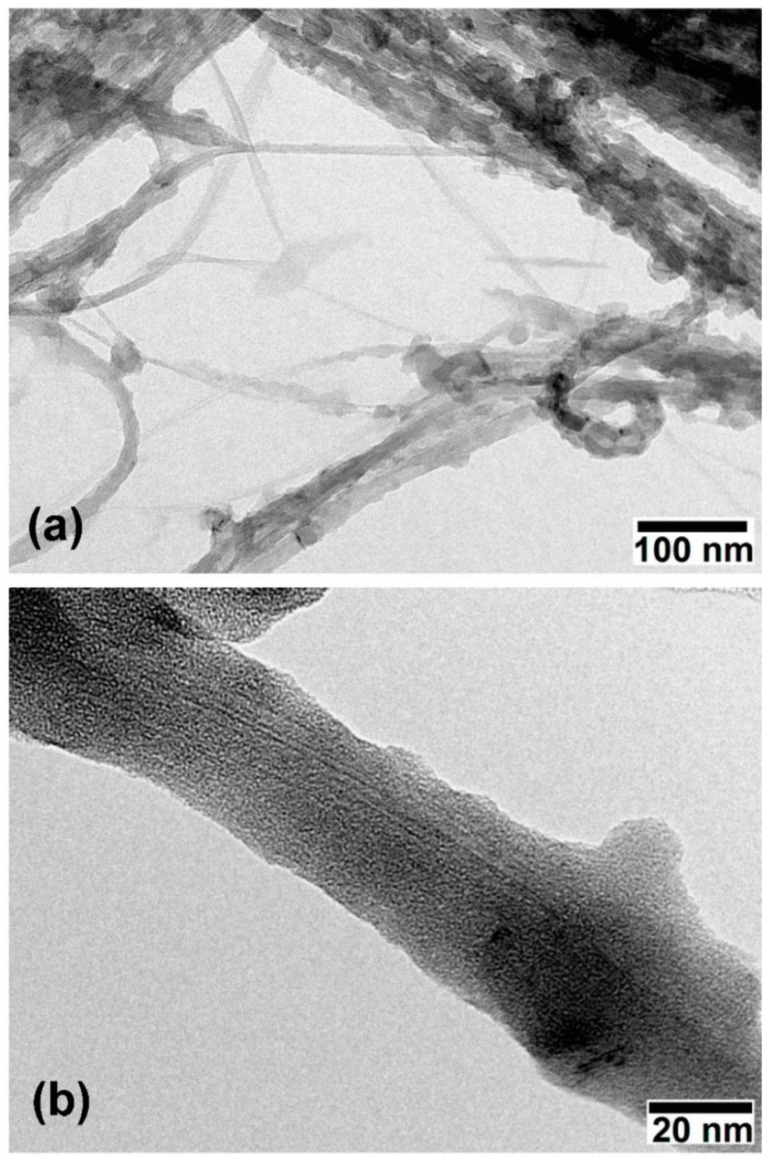
TEM images of CNTs–PAMAM–Ag. (**a**) revealed the efficient entanglement of CNTs by success-fully conjugating PAMAM chains onto their surfaces. (**b**) The PAMAM grafted to the surface of CNT and the deposition of spherical-shaped Ag nanoparticles.

The TEM image of CNTs–PAMAM–Ag (Figure 3a) revealed the efficient entanglement of CNTs by successfully conjugating PAMAM chains onto their surfaces. The CNTs present in CNTs–PAMAM–Ag have a diameter of a few nanometers and a length of several micrometers. The PAMAM grafted to the surface of CNT and the deposition of spherical-shaped Ag nanoparticles are distinctly visible in Figure 3b. The average particle size of Ag nanoparticles was around 35 nm. The PAMAM is efficiently grafted over the entire surface of CNTs. However, the thickness of PAMAM was not uniform. Additionally, the homogenous distribution of Ag nanoparticles and their firm adherence is visible in Figure 3. The coarse texture was created over the surface of CNTs–PAMAM–Ag, which enables the adsorption rate of pollutants. Additionally, the hydrophilic nature of PAMAM turns the hydrophobic CNTs into hydrophilic and increases the absorption proportion. The absence of Ag nanoparticles in the void space (Figure 3) reveals the strong adherence of Ag nanoparticles to the surface PAMAM grafted CNTs. The resilient immobilization of Ag nanoparticles over PAMAM grafted CNTs caused by strong interaction ensue between them, preventing Ag nanoparticles’ leaching.

**Figure 4:**
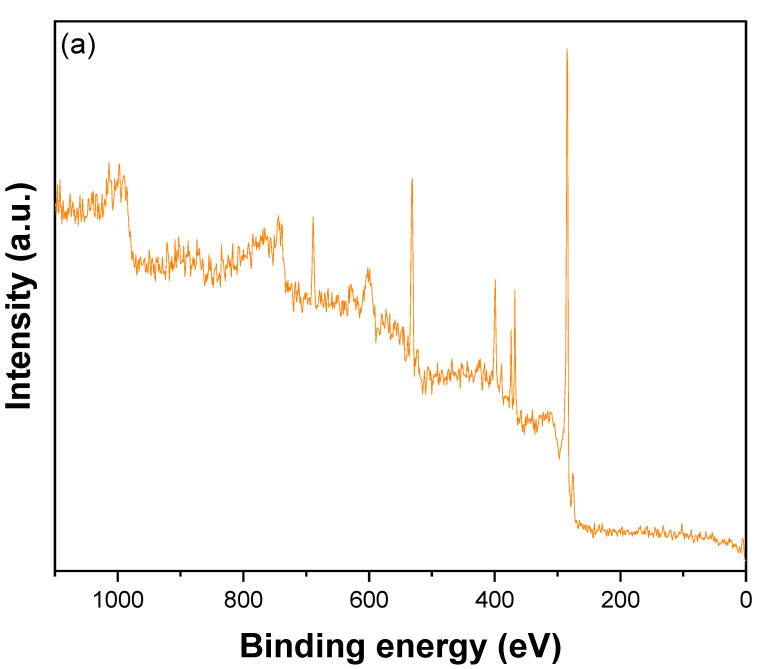

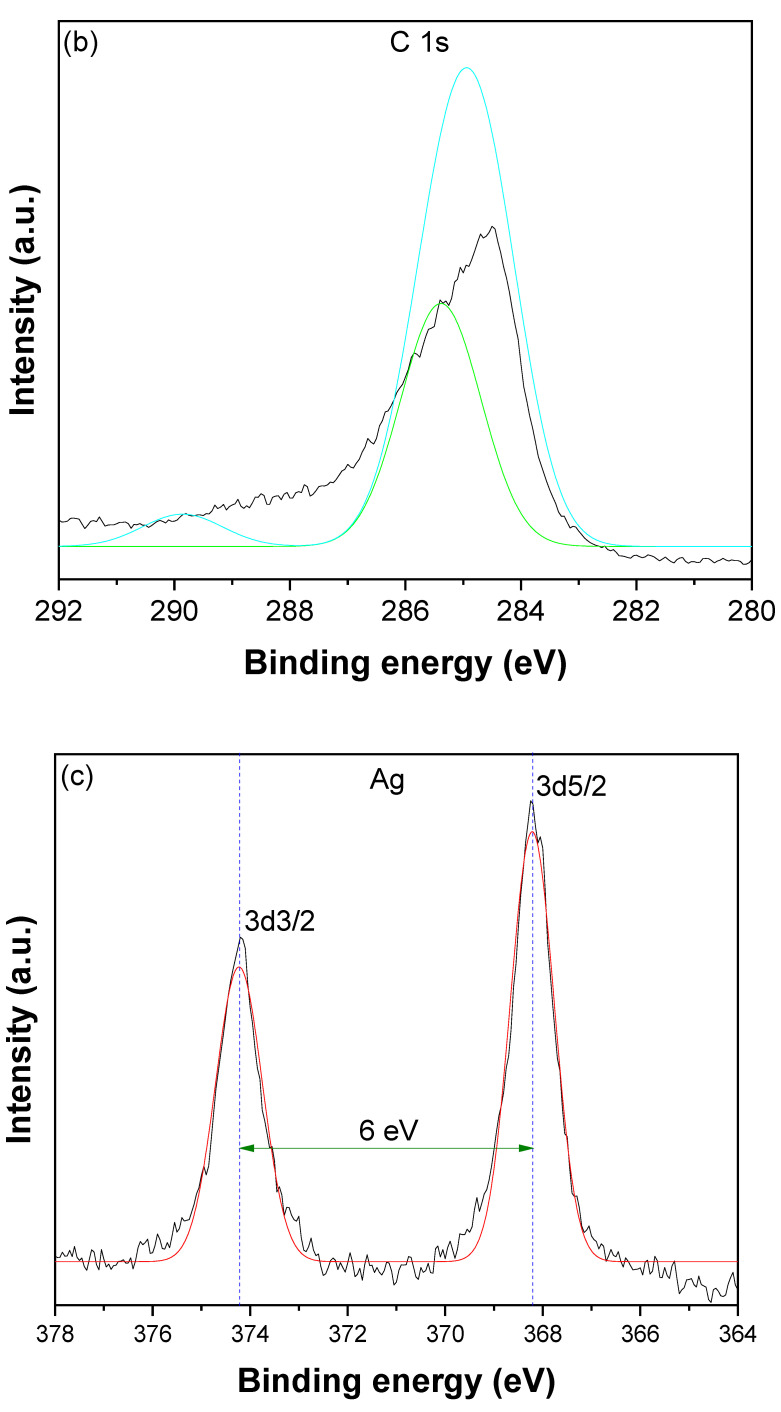

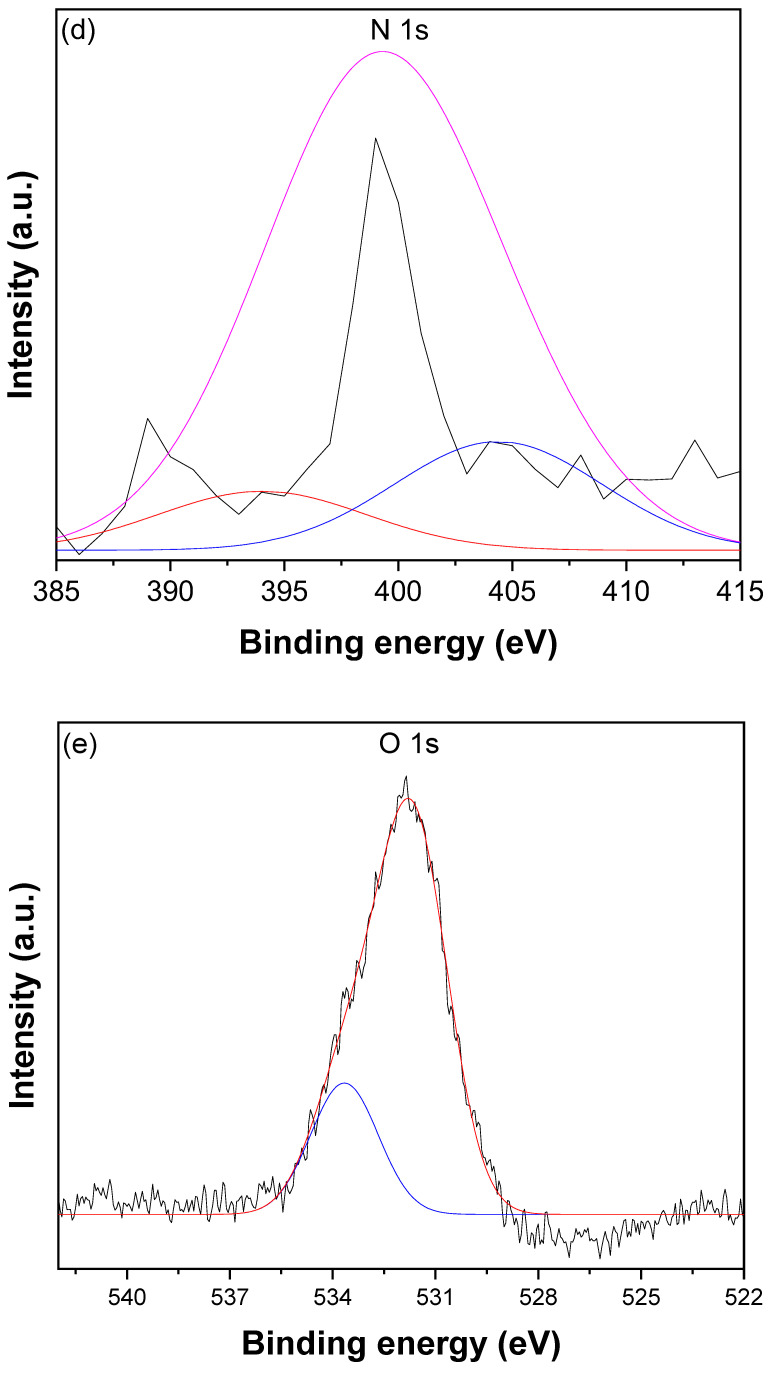
(**a**) XPS survey spectrum, (**b**) high-resolution spectrum of C1s, (**c**) high-resolution spectrum of Ag3d, (**d**) high-resolution spectrum of N 1s, and (**e**) high-resolution spectrum of O1s of CNTs–PAMAM–Ag.

The XPS survey spectrum of CNTs–PAMAM–Ag illustrated in Figure 4a confirms the presence of C, Ag, N, and O. The atomic ratio of Ag, C, and O estimated from XPS in CNTs–PAMAM–Ag was 1.17, 85.75, and 13.08%, respectively. The high-resolution spectrum of C1s (Figure 4b) exhibited a peak at 284.5 eV, divulge into three distinct peaks by Gaussian fitting. The peak at 284.9 eV was assigned to C-C bonds of sp^2^ hybridized carbon atoms of CNTs [59]. The peak at 285.4 eV was due to C-C bonds occurring in structurally defective sp^3^ hybridized carbon atoms and C=O bonds [59,60]. Another peak found at 290.0 eV was owing to carboxyl carbon O=C–O [61]. The high-resolution spectrum of Ag 3d (Figure 4c), disclosed into two peaks situated at 368.2 and 374.2 eV, featured the metallic state of Ag 3d5/2 and Ag 3d3/2, respectively [60]. The position of the peaks and binding energy match the value found for Ag^+^ [62,63]. The difference in the binding energy between Ag 3d5/2 and Ag 3d3/2 peaks was 6 eV [60]. It confirms the presence of Ag nanoparticles in the metallic state. The N 1s spectrum shown in Figure 4d, deconvoluted into three peaks located at 394.0 eV, 399.0 eV, and 405.0 eV corresponding to –N= (quinoid imine), –NH– (benzoid amine), and positively charged nitrogen(–HN^∙+^– and –HN^+^=), respectively [64]. The high-resolution spectrum of O 1s (Figure 4e) split into two peaks located at 530.1 eV by lattice O and 532.4 eV due to carbonyl (=C–O) functional groups [64].

**Figure 5:**
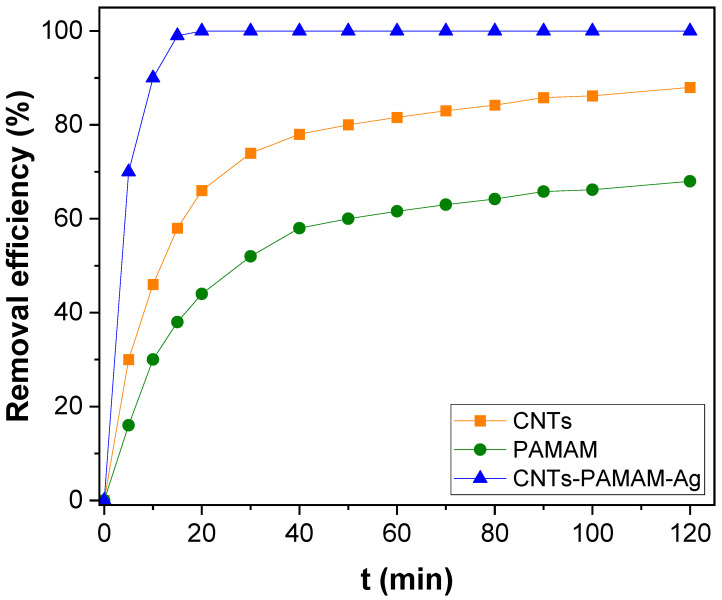
Kinetics of Pb(II) adsorption over oxidized CNTs, PAMAM and CNTs–PAMAM–Ag.

The absorption efficiency of CNTs–PAMAM–Ag was evaluated by estimating the adsorption rate of two important toxins in water, viz., Pb(II) and As(III). Initially, the adsorption ability of oxidized CNTs and PAMAM and its improvement by their conjugation after their conjugation and deposition of Ag nanoparticles was accessed by measuring the adsorption of Pb(II) as a function of contact time. The result found for adsorption as a function of contact time for 40 µg/L concentrated Pb(II) solution is shown in Figure 5. The adsorption performance of oxidized CNTs and PAMAM was significantly improved after their conjugation and deposition of Ag nanoparticles in CNTs–PAMAM–Ag. About 99% of Pb(II) was adsorbed by CNTs–PAMAM–Ag within 15 min, while it was 58 and 38% for oxidized CNTs and PAMAM, respectively. Further, to find the controlling mechanisms of the adsorption process and its dynamics, the pseudo-first, and the second-order kinetic models were applied using the following Equations (4) and (5).

Pseudo-first-order model:

(4) $$ln(q_{e}-q_{t})=lnq_{e}-k_{1}t$$

Pseudo-second-order model:

(5) $$\frac{t}{q_{t}}=\frac{1}{k_{2}\;q_{e}^{2}\;}+\frac{t}{q_{e}}$$

where *q_e_* and *q_t_* are the quantity of adsorbate (mg/g) at equilibrium and particular time *t* (min), respectively; *k*_1_ (min^−1^) and k_2_ [g/(mg.min)] are the pseudo-first and second-order rate constants, respectively.

**Figure 6:**
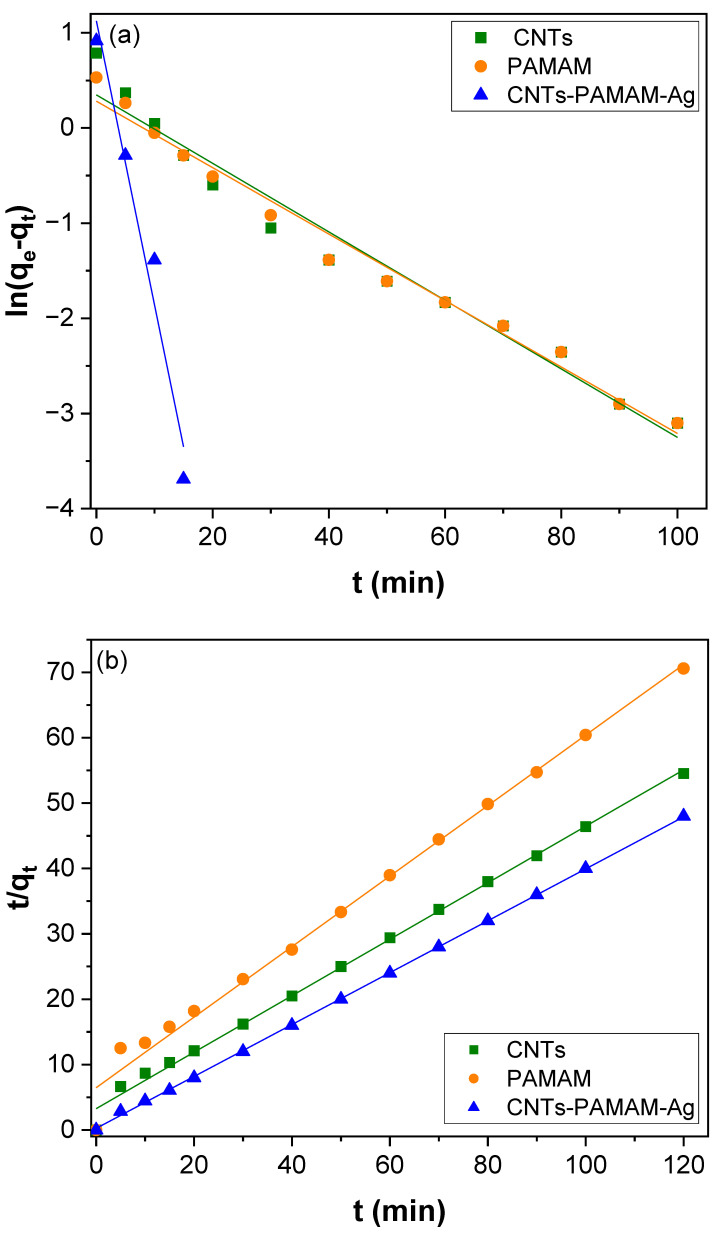
(**a**) Pseudo-first-order and (**b**) pseudo-second-order kinetics for Pb(II) adsorption over oxidized CNTs, PAMAM and CNTs–PAMAM–Ag.

**Figure 7:**
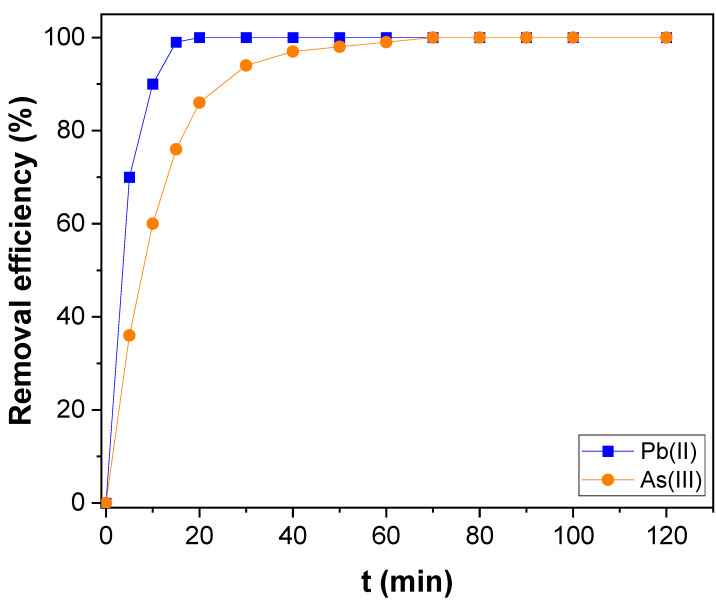
Kinetics of Pb(II) and As(III) adsorption over CNTs–PAMAM–Ag.

**Figure 8:**
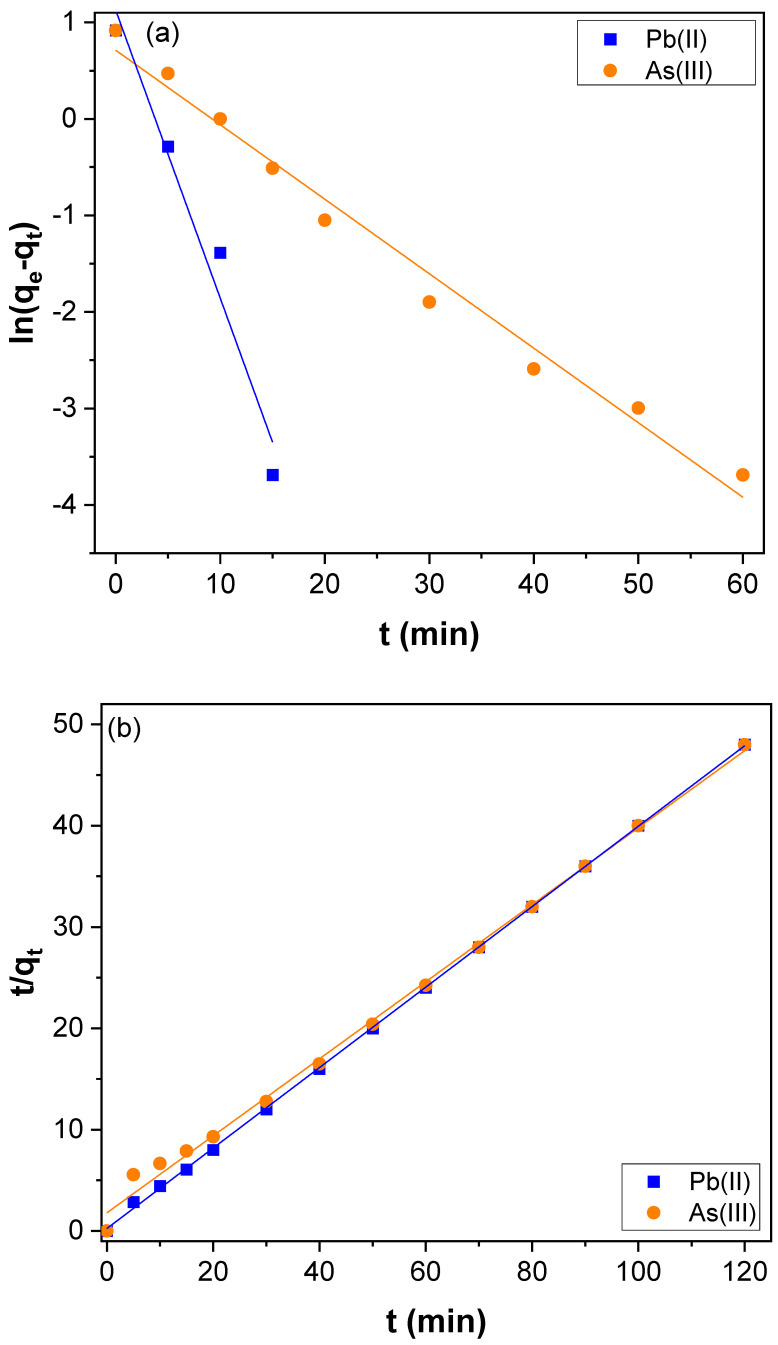
(**a**) Pseudo-first-order and (**b**) pseudo-second-order kinetics for Pb(II) and As(III) adsorption over CNTs–PAMAM–Ag.

Figure 6a is the pseudo-first-order plot got for the adsorption of Pb(II) of over-oxidized CNTs, PAMAM, and CNTs–PAMAM–Ag, and Figure 6b is acquired for pseudo-second-order kinetics. The parameters estimated are illustrated in Table 1. The correlation coefficient (R^2^) received for pseudo-second-order kinetics was higher than the value estimated for the pseudo-first-order kinetics. In addition, the value of *q_e_* (exp) agreed with *q_e_* (cal) determined from pseudo-second-order kinetics rather than the value of pseudo-first-order kinetics. Therefore, the adsorption of Pb(II) over oxidized CNTs, PAMAM, and CNTs–PAMAM–Ag takes place through pseudo-second-order kinetics. Further, the adsorption rate of Pb(II) over CNTs–PAMAM–Ag was compared with the adsorption of As(III). The kinetics profile found for the adsorption of Pb(II) and As(III) is illustrated in Figure 7 and the plot perceived for q_t_ versus time is given in Figure S1. It was revealed that the adsorption of Pb(II) and As(III) is time-dependent and proceeds as a function of time. Consequently, contact time is an essential factor in the adsorption of Pb(II) and As(III) and plays a significant role. The behavior in the adsorption of both Pb(II) and As(III) was identical. The adsorption rate was significant in the initial stage, and the progression in the later phase was relatively slow until the attainment of equilibrium. Within 15 min, about 99% of Pb(II) and 76% of As(III), was adsorbed. To reach the equilibrium, 20 and 70 min was needed for Pb(II) and As(III), respectively. After conquering the equilibrium, the adsorption of Pb(II) and As(III) was minute until the measured period of 120 min. The rapid adsorption in the initial stage could be due to the abundant availability of the active sites existing over the surface of CNTs–PAMAM–Ag. With the progress of time, the active sites are being saturated by the adsorption of a high number of Pb(II) and As(III) ions [65]. In addition, the repulsive forces befall the solute molecules in the solid and bulk phases [65]. The pseudo-first and pseudo-second-order plots received for adsorption of Pb(II) and As(III) over CNTs–PAMAM–Ag are demonstrated in Figure 8a,b, respectively, and the related parameters assessed are summarized in Table 1. The R^2^ value for pseudo-first-order kinetics was 0.9663 and 0.9845 for Pb(II) and As(III), respectively. For pseudo-second-order kinetics, it was 0.9998 and 0.9966 for Pb(II) and As(III), respectively. The R^2^ value for pseudo-second-order kinetics is higher than that of pseudo-first-order kinetics. Hence, the adsorption of Pb(II) and As(III) could be well-fitted with the pseudo-second-order kinetic model. Therefore, during adsorption, chemisorption ensues by sharing or exchanging of electrons between Pb(II) or As(III) ions and CNTs–PAMAM–Ag, which could be the rate-controlling step in the process of adsorption [5,66]. It is presumed that the pseudo-second-order adsorption process ensues through surface reactions until active sites get saturated, followed by the incidence of diffusion for complex sequential interactions [5,67].

**Figure 9:**
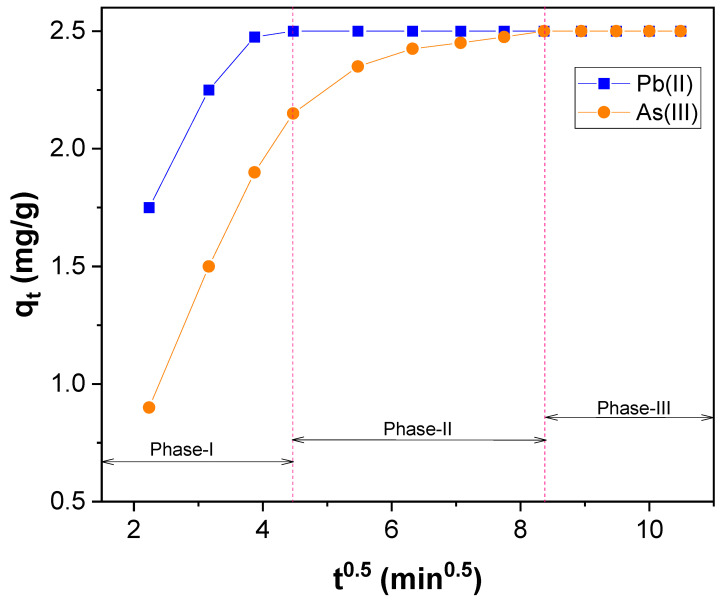
Weber-Morris intraparticle diffusion plot for Pb(II) and As(III) adsorption over CNTs–PAMAM–Ag.

To find the reaction pathways and the rate-controlling step in adsorption, Weber−Morris intraparticle pore diffusion model was used [68]. This model is based on sorbate species transport into the sorbent’s pore is often the rate-controlling step in the adsorption. Thus, rate constants for intraparticle diffusion (*k_id_*) were estimated using Equation (6).

(6) $$q_{t}=k_{id}\;t^{0.5}+c$$

where *q_t_* (mg/g) is the amount of Pb(II) and As(III) adsorbed at time t (min); *c* (mg/g) is the intercept that represents the boundary layer effect, and *k_id_* [mg/(g.min^0.5^)] is the intraparticle diffusion rate constant, which can be evaluated from the slope of the linear intraparticle diffusion plot of *q_t_* versus *t*^0.5^ (Figure 9 and Figure S2). If the regression of the intraparticle diffusion plot is linear and passes through the origin, in that case, intraparticle diffusion is the sole rate-limiting step in the adsorption. However, the plot of *q_t_* versus *t*^0.5^, shown in Figure 9, does not pass through the origin (Figure S2). So, intraparticle diffusion is not the sole rate-limiting step in the adsorption found for Pb(II) and As(III). Moreover, if the intercept of the intraparticle diffusion plot is significant, the contribution of the surface sorption is more efficient in the rate-controlling step [69]. Contradictorily, the value of the intercept appraised for the adsorption of Pb(II) and As(III) was small (Table S1). In addition, the intraparticle diffusion plot (Figure S2) unveiled two straight lines. This specifies that intraparticle diffusion was not exclusively a rate-controlling step in the adsorption of Pb(II) and As(III); instead, it was regulated by both intraparticle diffusion and boundary layer effect [69,70]. The multilinearity found for the intraparticle diffusion plot (Figure 9) reveals that the adsorption of Pb(II) and As(III) emerge through multiple phases instead of a single [5]. Out of these, the first phase was owing to the instantaneous adsorption of Pb(II) and As(III) ions (Figure 9), and the second phase was due to the diffusion of Pb(II) and As(III) ions into the pores of CNTs–PAMAM–Ag. Finally, the third phase was from the equilibrium of adsorption that causes chemical reaction/bonding [5].

**Figure 10:**
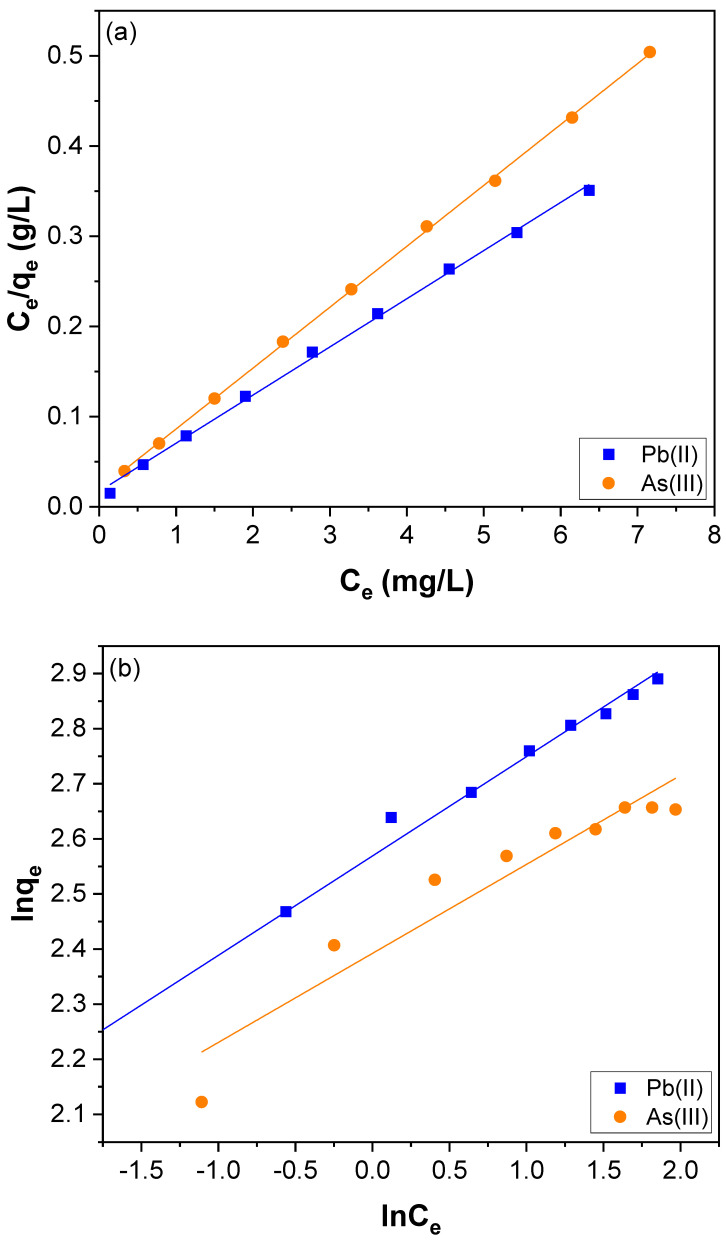

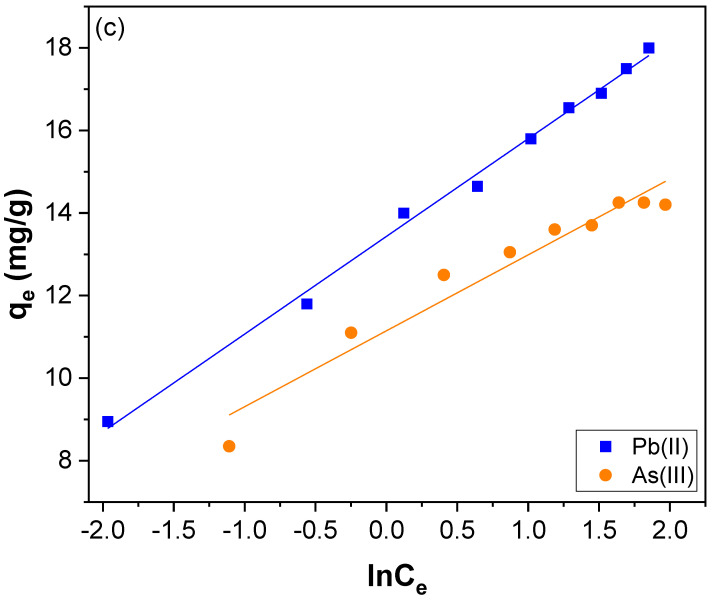
(**a**) Langmuir, (**b**) Freundlich, and (**c**) Temkin isotherms for Pb(II) and As(III) adsorption over CNTs–PAMAM–Ag.

To further explore the adsorption equilibrium, the experimental results obtained for adsorption of Pb(II) and As(III) were analyzed with three isotherm models viz., the Langmuir, the Freundlich, and the Temkin models. The Langmuir isotherm model assumes that monolayer adsorption occurs on the surface of the adsorbent [71]. Additionally, it presumes that the equivalent binding site number is specific and that adsorbate does not transmigrate [72]. Accordingly, the Langmuir model is represented by Equation (7).

(7) $$\frac{C_{e}}{q_{e}}=\frac{C_{e}}{q_{m}}+\frac{1}{K_{L\;}q_{m}}\;$$

where *q_e_* (mg/g) is the amount of adsorbed Pb(II) and As(III) per unit mass of CNTs–PAMAM–Ag; *C_e_* (mg/L) is the concentration of Pb(II) and As(III) at equilibrium; *q_m_* is the maximum amount of the Pb(II) and As(III) adsorbed per unit mass of CNTs–PAMAM–Ag to form a complete monolayer on the surface-bound at high *C_e_*. *K_L_* is the Langmuir adsorption constant related to the free energy of adsorption. The linear fitting for the Langmuir plot of specific adsorption (*C_e_*/*q_e_*) versus the equilibrium concentration (*C_e_*) is shown in Figure 10a. The parameters calculated by the Langmuir isotherm model are tabulated in Table 2. The maximum adsorption capacity (*q_m_*) calculated for the adsorption of Pb(II) and As(III) was 18.7 and 14.8 mg/g, respectively.

Furthermore, the kinetic data of adsorption of Pb(II) and As(III) was explored with the Freundlich isotherm model that defines the heterogeneous adsorption using Equation (8) [73].

(8) $$ln\;q_{e}=ln\;K_{F}+\frac{ln\;C_{e}}{n}\;$$

where *q_e_* (mg/g) is the amount of Pb(II) and As(III) adsorbed per unit mass of CNTs–PAMAM–Ag; *C_e_* (mg/L) is the concentration of Pb(II), and As(III) at the equilibrium, *K_F_* indicates the affinity of adsorbent of Pb(II) and As(III) and *n* denotes the adsorption intensity. *C_e_* (mg/L) is the concentration of Pb(II) and As(III) at equilibrium. The Freundlich isotherm fitting plot is shown in Figure 10b, and obtained results are summarized in Table 2. The magnitude of the exponent, 1/*n*, indicates the favorability of adsorption. The value of *n* ranging from 1 to 10 indicates the favorable conditions for adsorption [66]. The value of *n* calculated for Pb(II) and As(III) adsorption were 5.546 and 6.192, respectively. This represents that the adsorption of Pb(II) and As(III) over CNTs–PAMAM–Ag is favorable.

Apart, the adsorption of Pb(II) and As(III) was explored with the Temkin isotherm model [74] using Equation (9).

(9) $$\;q_{e}=B\;lnA+B\;ln\;C_{e}$$

where *B* = RT/*K_T_*, *K_T_* is the Temkin constant related to the heat of adsorption (J/mol); *A* is the Temkin isotherm constant (L/g), R is the gas constant (8.314 J/mol K), and T the absolute temperature (K). The Temkin isotherm fitting plot of *q_e_* versus ln *C_e_* is shown in Figure 10c, and the estimated parameters are listed in Table 2.

Among all the isotherm models, the Langmuir isotherm model was well suited for the adsorption of Pb(II) and As(III) (Figure 10a) with the R^2^_Lan_ value of 0.9980 and 0.9997 for Pb(II) and As(III), respectively. Nonetheless, it was lower for both the Freundlich and Temkin isotherm models. It means R^2^_Lan_ was high compared to R^2^_Fre_ and R^2^_Tem_. Thus, the experimental kinetic data received for Pb(II) and As(III) adsorption was well fitted to the Langmuir isotherm model. So the Langmuir isotherm model satisfactorily explains the adsorption of Pb(II) and As(III) compared to the Freundlich and Temkin isotherm models. As the Langmuir equation assumes the adsorbent’s surface is homogenous, a better fitting of the Langmuir isotherm model indicates the uniform distribution of active sites over the entire surface of CNTs–PAMAM–Ag and their homogeneity [66,69]. It signifies that the adsorption of Pb(II) and As(III) was dominated by the monolayer binding on the homogeneous surface of CNTs–PAMAM–Ag [66]. The maximum adsorption capacity (*q_m_*) of CNTs–PAMAM–Ag estimated for Pb(II) and As(III) adsorption was compared to the value reported for adsorbents, which is listed in Table S2 [4,42,75,76,77,78,79,80,81,82].

Further, to investigate the effect of pH on the adsorption of Pb(II) monitored at pH of 4, 6, 8, 10, and 12. It was found that the adsorption of Pb(II) was on the pH of the solution in such a way that the maximum adsorption was perceived at around 8 (Figure S3). However, the adsorption rate was low at other measured values of pH viz., 4, 6, 10, and 12. The natural pH of the 40 μg/L Pb(II) solution used in the adsorption measurement was 8 (7.67). So the adsorption at pH 8 was recorded with an alteration of pH by adding either 0.1 M HCl or NaOH. However, the adsorption measurement at other pH values such as 4 and 6 was adjusted using 0.1 M HCl, while pH 10 and 12 were attained by adding 0.1 NaOH. Hence, the addition of HCl or NaOH could interfere with Pb(II) ions and hinder the adsorption efficiency. The recovery and repeated use of the adsorbent are essential for practical application. The efficiency of CNTs–PAMAM–Ag in the adsorption of Pb(II) was investigated for four successive cycles (Figure S4). The adsorption rate of Pb(II) was not reduced significantly in all four studied cycles. Therefore, CNTs–PAMAM–Ag is an ideal adsorbent for repeated use without losing its activity. The hierarchical architecture generated grafting of PAMAM improved the adsorption efficiency of CNTs, which was further enhanced by the deposition of Ag nanoparticles.

## 4. Conclusions

In conclusion, an efficient adsorbent for effective adsorption of Pb(II) and As(III) was successfully prepared by grafting fourth-generation aromatic PAMAM to CNTs and successive deposition of Ag nanoparticles. Thus produced CNTs–PAMAM–Ag was able to adsorb 99 and 76% of Pb(II) and As(III), respectively within 15 min. The kinetics data obtained for the adsorption of Pb(II) and As(III) was well fitted with the pseudo-second-order model compared to the pseudo-first-order model. It revealed the occurrence of chemisorption by sharing or exchanging electrons between Pb(II) or As(III) ions and CNTs–PAMAM–Ag. It could be the rate-controlling step in the process of adsorption. The multilinearity of the Weber−Morris plot demonstrated that intraparticle diffusion was not a rate-controlling step in the adsorption of Pb(II) and As(III); instead, it was regulated by both intraparticle diffusion and boundary layer effect. The proper fitting of kinetic data of Pb(II) and As(III) adsorption with the Langmuir isotherm model indicates the uniform distribution of active sites over the entire surface of CNTs–PAMAM–Ag and their homogeneity. In addition, it signifies that the adsorption of Pb(II) and As(III) was dominated by the monolayer binding on the homogeneous surface of CNTs–PAMAM–Ag. The adsorption ability of CNTs–PAMAM–Ag depends on the pH. The CNTs–PAMAM–Ag is an ideal adsorbent for repeated use without losing its activity. Because of its significance in Pb(II) and As(III) adsorption, CNTs–PAMAM–Ag could be an efficient adsorbent and practically applicable for the adsorption of other heavy metals and other contaminants present in water.

## Figures and Tables

**Table 1:** Parameters calculated from pseudo-first, and second-order kinetic models.

Adsorbent		*q_e_* (exp) mg/g	Pseudo-First Order Kinetic Model			Pseudo-Second-Order Kinetic Model		
	Adsorbate		*q_e_* (cal) mg/g	*k*_1_ min^−1^	R^2^	*q_e_* (cal) mg/g	*k*_2_ g.mg^−1^.min^−1^	R^2^
CNTs	Pb(II)	2.2	0.3483	0.0360	0.9687	2.3152	0.0573	0.9960
PAMAM	Pb(II)	1.7	0.2815	0.0349	0.9847	1.8543	0.0449	0.9896
CNTs–PAMAM–Ag	Pb(II)	2.5	1.1255	0.2983	0.9663	2.5189	0.6425	0.9993
CNTs–PAMAM–Ag	As(III)	2.5	0.7117	0.0772	0.9845	2.6309	0.0806	0.9966

**Table 2:** Parameters calculated from Langmuir, Freundlich, and Temkin adsorption isotherms.

Adsorbate	Langmuir Isotherm			Freundlich Isotherm			Temkin Isotherm		
	*q_m_* mg/g	*K_L_* L/mg	R^2^_Lan_	*K_F_* mg/g	*n*	R^2^_Fre_	*A*	*B*	R^2^_Tem_
Pb(II)	18.7	3.12	0.9980	13.05	5.546	0.9916	292.3	2.4	0.9950
As(III)	14.8	3.59	0.9997	10.94	6.192	0.9100	428.9	6.1	0.9451

## Data Availability

Not applicable.

## Supplementary Materials

The following supporting information can be downloaded at: https://www.mdpi.com/article/10.3390/nano12213852/s1. Figure S1: Plot perceived qt as a function of time for Pb(II) and As(III) adsorption over CNTs–PAMAM–Ag; Figure S2: Intraparticle diffusion model for Pb(II) and As(III) adsorption over CNTs–PAMAM–Ag; Figure S3: Effect of pH on the adsorption of Pb(II) over CNTs–PAMAM–Ag; Figure S4: Efficiency of CNTs–PAMAM–Ag in the adsorption of Pb(II) for four successive cycles. Table S1: Parameters calculated from the intra-particle diffusion plot provided in Figure S2; Table S2: Comparison of maximum adsorption capacity (qm) of CNTs–PAMAM–Ag estimated for Pb(II) and As(III) adsorption with different adsorbents.
