# Supplementary material for: Elevated Adsorption of Lead and Arsenic over Silver Nanoparticles Deposited on Poly(amidoamine) Grafted Carbon Nanotubes
Source: Nanomaterials (Basel). 2022 Nov 1;12(21):3852. doi: 10.3390/nano12213852 (PMC9654323; doi:10.3390/nano12213852)
Supplement: Supplementary file 1 [file nanomaterials-12-03852-s001.zip › nanomaterials-1960567-Supplementary.pdf]

Supplementary Material

Elevated Adsorption of Lead and Arsenic over Silver Nanoparticles Deposited on Poly(amidoamine) Grafted Carbon Nanotubes

Gururaj M. Neelgund [1,*], Sanjuana F. Aguilar [1], Mahaveer D. Kurkuri [2], Debora F. Rodrigues [3] and Ram L. Ray [4]

1 Department of Chemistry, Prairie View A&M University, Prairie View, TX 77446, USA
2 Centre for Research in Functional Materials (CRFM), JAIN University, Jain Global Campus, Bengaluru 562112, Karnataka, India
3 Department of Civil and Environmental Engineering, University of Houston, Houston, TX 77004, USA
4 College of Agriculture and Human Sciences, Prairie View A&M University, Prairie View, TX 77446, USA
* Correspondence: gmneelgund@pvamu.edu

Table S1: Parameters calculated from the intra-particle diffusion plot provided in Figure S2.

Adsorbent	Intra-particle diffusion model					
	K_{id-1} mg/(g.min$^{0.5}$)	c	R^2	k_{id-2} mg/(g.min$^{0.5}$)	c	R^2
Pb(II)	0.6587	0.0920	0.9797	1.32x10^{-16}	2.5000	-
As(III)	0.4874	-0.0547	0.9873	0.0472	2.0649	0.7141

Table S2: Comparison of maximum adsorption capacity (q_m) of CNTs–PAMAM–Ag estimated for Pb(II) and As(III) adsorption with different adsorbents.

**Adsorbent**	**Adsorbate**	**maximum adsorption capacity q_m (mg/g)**	**pH**	**Ref**
CNTs–PAMAM–Ag	Pb(II)	18.7	7.7	This work
6O-MWCNTs@Fe_3O_4	Pb(II)	215.05	6.0	42
MWCNTs/ThO_2	Pb(II)	178.25	5.5	75
50%CNTs/Fe_3O_4	Pb(II)	40.88	6.2	76
CNTs	Pb(II)	102.04	5.0	77
CS-MA-DETA	Pb(II)	239.2	5.0	78
CNTs–PAMAM–Ag	As(III)	14.8	8.1	This work
HDTMA-Al-bentonite	As(III)	2.24	4.5	79
Iron-impregnated charred GAP	As(III)	3.25	7.0	80
Aluminum pillared HDTMA sericite	As(III)	0.40	4.5	81
CTMAB-Fe-Montmorillonite	As(III)	11.36	6.5	82
Fe/Mn-HDTMA kaolin	As(III)	7.99	6.5	4

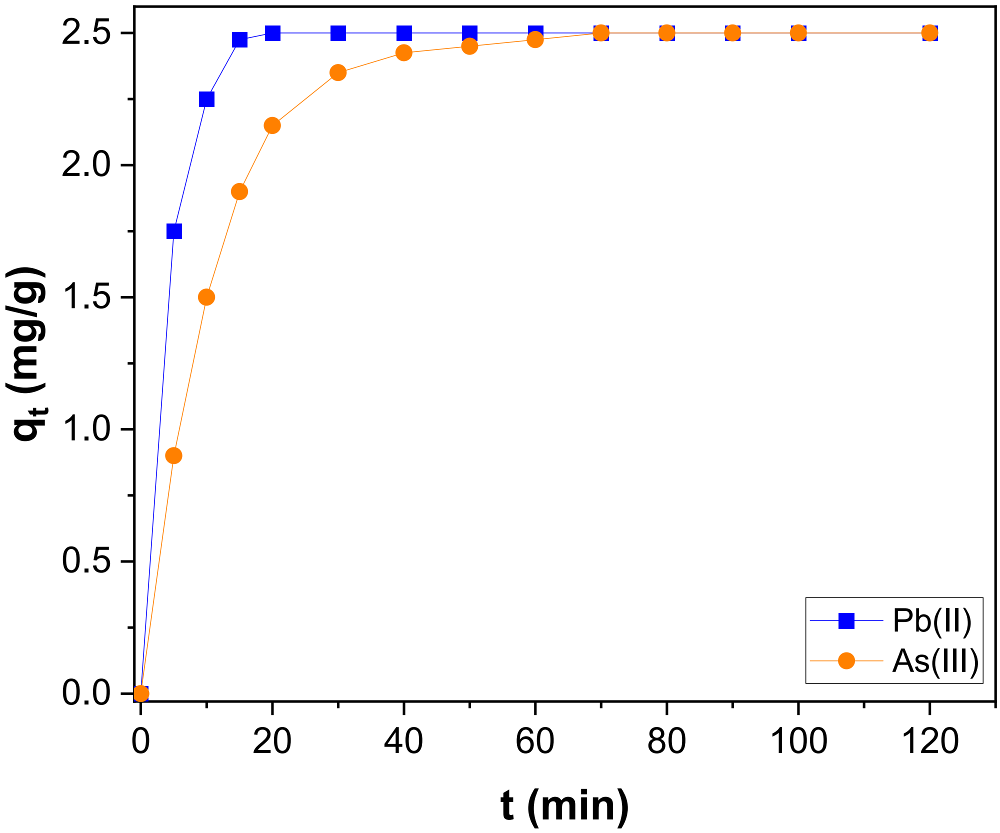

Figure S1: Plot perceived q_t as a function of time for Pb(II) and As(III) adsorption over CNTs–PAMAM–Ag.

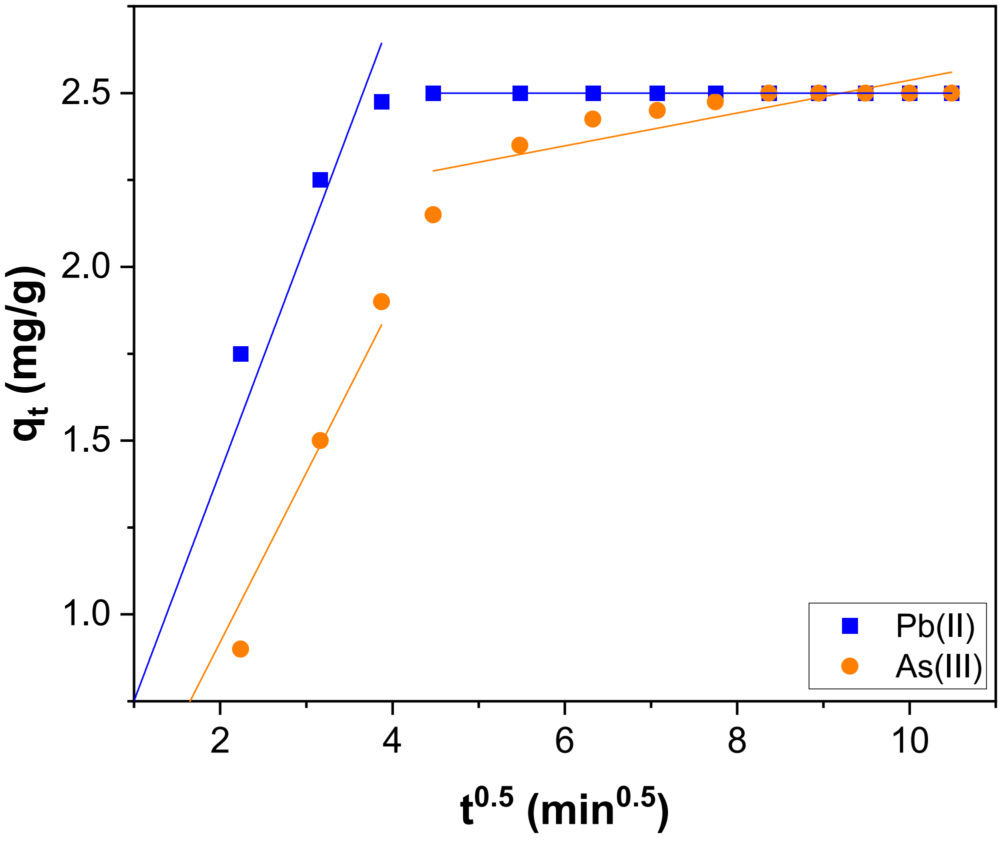

Figure S2: Intraparticle diffusion model for Pb(II) and As(III) adsorption over CNTs–PAMAM–Ag.

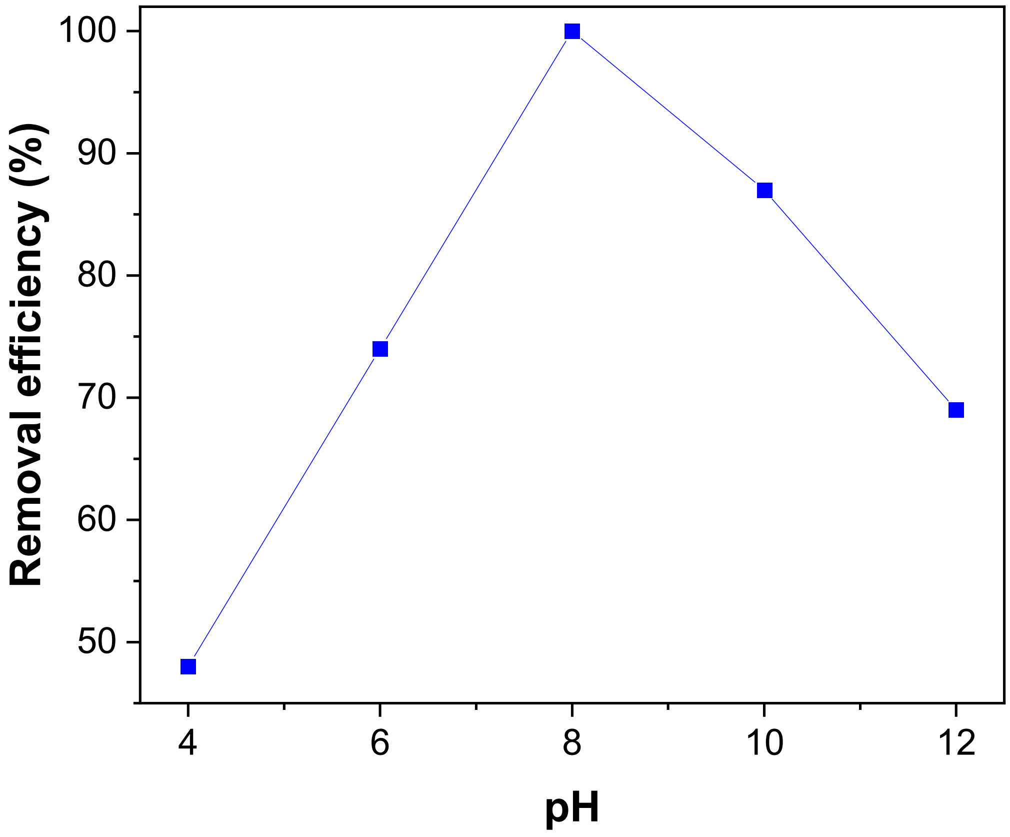

Figure S3: Effect of pH on the adsorption of Pb(II) over CNTs–PAMAM–Ag.

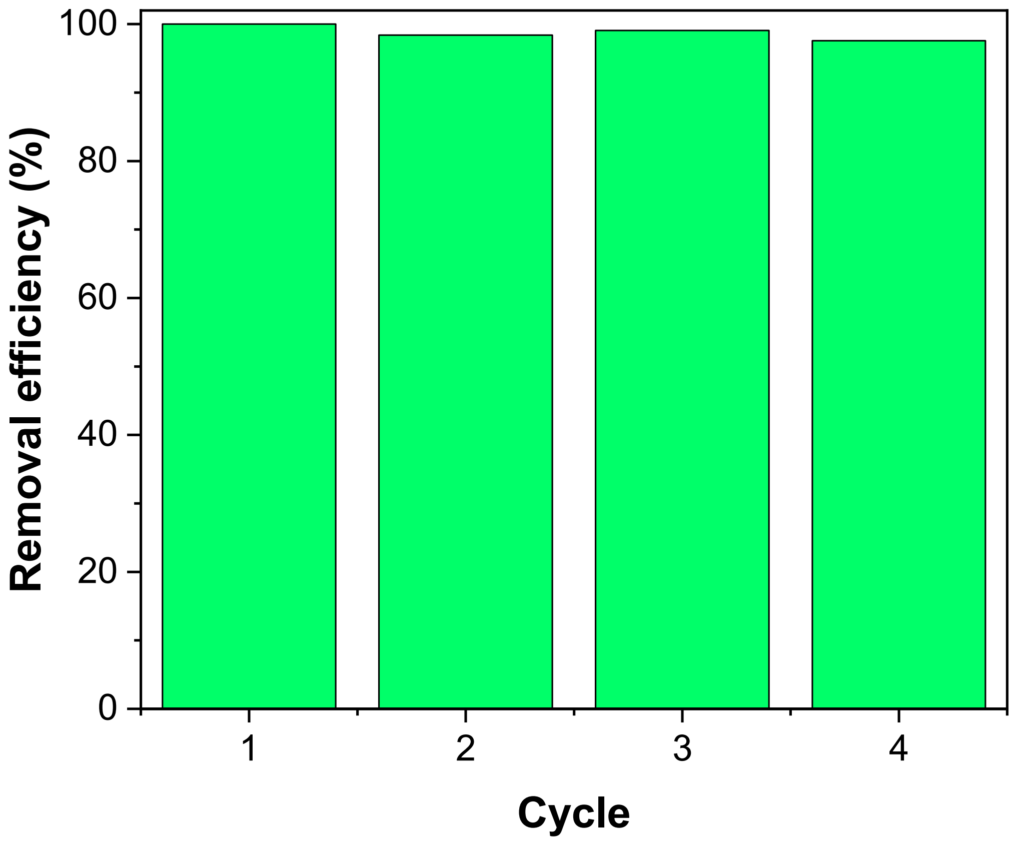

Figure S4: Efficiency of CNTs–PAMAM–Ag in the adsorption of Pb(II) for four successive cycles.
